# Radiation hardness of cadmium telluride solar cells in proton therapy beam mode

**DOI:** 10.1371/journal.pone.0221655

**Published:** 2019-09-12

**Authors:** Shinhaeng Cho, Sang Hee Ahn, Ick Joon Cho, Yong Hyub Kim, Jae-Uk Jeong, Mee Sun Yoon, Sung-Ja Ahn, Woong-Ki Chung, Taek-Keun Nam, Ju-Young Song

**Affiliations:** 1 Department of Radiation Oncology, Chonnam National University Medical School, Gwangju, Korea; 2 Proton Therapy Center, National Cancer Center, Goyang, Korea; University of California Santa Barbara, California, USA

## Abstract

We evaluated the durability of cadmium telluride (CdTe) solar cells upon proton beam irradiation as well as the possibility of achieving a dosimeter usable in proton beam therapy by applying 100 MeV of pencil beam scanning (PBS) irradiation. Specifically, a 100 MeV proton PBS beam was applied at irradiation doses of 0, 10^12^, 10^13^, 10^14^, and 10^15^ cm^-2^. According to the results, the remaining factors (defined as the ratio of the degraded value to the initial value) of open-circuit voltage (*V*_*oc*_), short-circuit current (*J*_*sc*_), fill-factor (*FF*), and efficiency (*ƞ*) which are solar cell performance parameters, were approximately 89%, 44%, 69%, and 30%, respectively, compared to those of the reference cell (without irradiation) at the highest dose of 1×10^15^ cm^-2^. In particular, the conversion efficiency, which is the main factor, was approximately 70% of that of the reference cell even at a high fluence of 1×10^14^ cm^-2^. In addition, we observed the projected range of the hydrogen atoms based on the PBS beam energy using the Tool for Particle Simulation software and assessed the amount of fluence accumulated in a CdTe cell. As the energy increased, the fluence accumulated inside the cell tended to decrease owing to the characteristics of the Bragg peak of the proton. Thus, the radiation damage to the cell induced by the proton beam was reduced. The results of this study are expected to provide valuable reference information for research on dosimetry sensors composed of thin-film solar cells, serving as the basis for future application in proton beam therapy with CdTe solar cells.

## Introduction

The radiation hardness of solar cells has been an important issue in space missions. Solar cells experience harsh radiation during missions, where particles such as protons and electrons cause severe performance and durability degradation [1]. There have been numerous studies on the performance deterioration of various types of photovoltaic detectors, such as organic photovoltaics, cadmium telluride (CdTe) cells, Perovskite solar cells, GaInP/GaInAs/Ge triple junction solar cells, and copper indium gallium selenide cells due to irradiation by protons and electrons [2–6]. Among other types of thin film solar cells, CdTe-based solar cells are suitable for space missions owing to their economic advantages, relatively simple process, and high specific power relative to their thinness [7,8]. In addition, the properties for process optimization and improvement of efficiency are being studied [9,10].

Two studies were recently conducted on the performance degradation of CdTe devices after irradiation depending on the change in proton flux [11,12]. Yang et al. investigated the performance degradation owing to a change in fluence by irradiating CdTe cells with a 15 MeV proton beam [11]. Lamb et al. assessed the performance parameters of CdTe cells fabricated on a cerium-doped cover glass, which is more durable than conventional borosilicate glass, and irradiated them with a 0.1 MeV proton beam [12]. Based on the above studies, we predicted that the performance degradation of solar cells through proton irradiation depends on the energy fluence rather than the energy value.

In this report, we discuss not only the radiation hardness of CdTe solar cells, but also their usability in proton beam dosimetry within the radiation therapy beam range. Thin and flexible photovoltaic-based cells can be fabricated at low cost compared to conventional solid-state detectors, making them suitable for use as thin-film array dosimetry sensors or large-area portal imaging sensors [13–16]. In proton beam dosimetry, semiconductor-based devices require materials that convert signals into different forms because the energy is generally too strong for direct signals to be received, or a buffer layer is applied in front of the absorber layer to shorten the energy transmission length. Parsai et al. developed a CdTe thin film detector using a metal plate to convert a 6 MV photon beam into secondary electrons and then conducted a study on detecting a 6 MV therapy beam [17]. The results of 6 MV percentage depth dose measurement using CdTe cells agreed well with those of a Monte Carlo simulation in an ion chamber. Jang et al. measured the spread-out Bragg peak of a proton scattering beam using an optical fiber-based sensor and confirmed that it matched the ion chamber results by correcting the quenching effect caused by the scintillator material [18]. Son et al. showed that the Bragg peak of a pencil beam-shaped scanning proton beam could be measured without quenching using a fiber-optic array sensor without a scintillator material [19]. These optical fiber-based sensors use an indirect method that converts proton radiation into visible light and then into an electrical signal using a photomultiplier tube; thus, the signal processing is complex. Because it is difficult to commercialize array detectors, the need for a semiconductor-based dosimetry sensor has been continuously emphasized. To use a solar cell as a proton beam dosimetry sensor, it is necessary to investigate the absorption, transmission, and performance degradation of the cells in the proton therapy beam range.

We investigated the solar cell performance degradation within the energy range of proton therapy beams, namely, above 100 MeV. Although there have been different reports on solar cell degradation due to proton beam irradiation, this report is the first to address solar cell performance within the energy range of proton therapy beams. We also discuss the possibility of using solar cells as tools for proton therapy dosimetry in proton therapy mode.

Specifically, we observed the performance degradation of CdTe solar cells when protons with energies of 100 MeV or more were applied. It was confirmed that the performance deterioration of solar cells during proton beam irradiation is determined by the fluence rather than the energy level. In addition, the dependence of the penetration depth of a proton beam on the energy level was determined through a Monte Carlo simulation.

## Materials and methods

### Proton therapy beam irradiation

**Fig 1:**
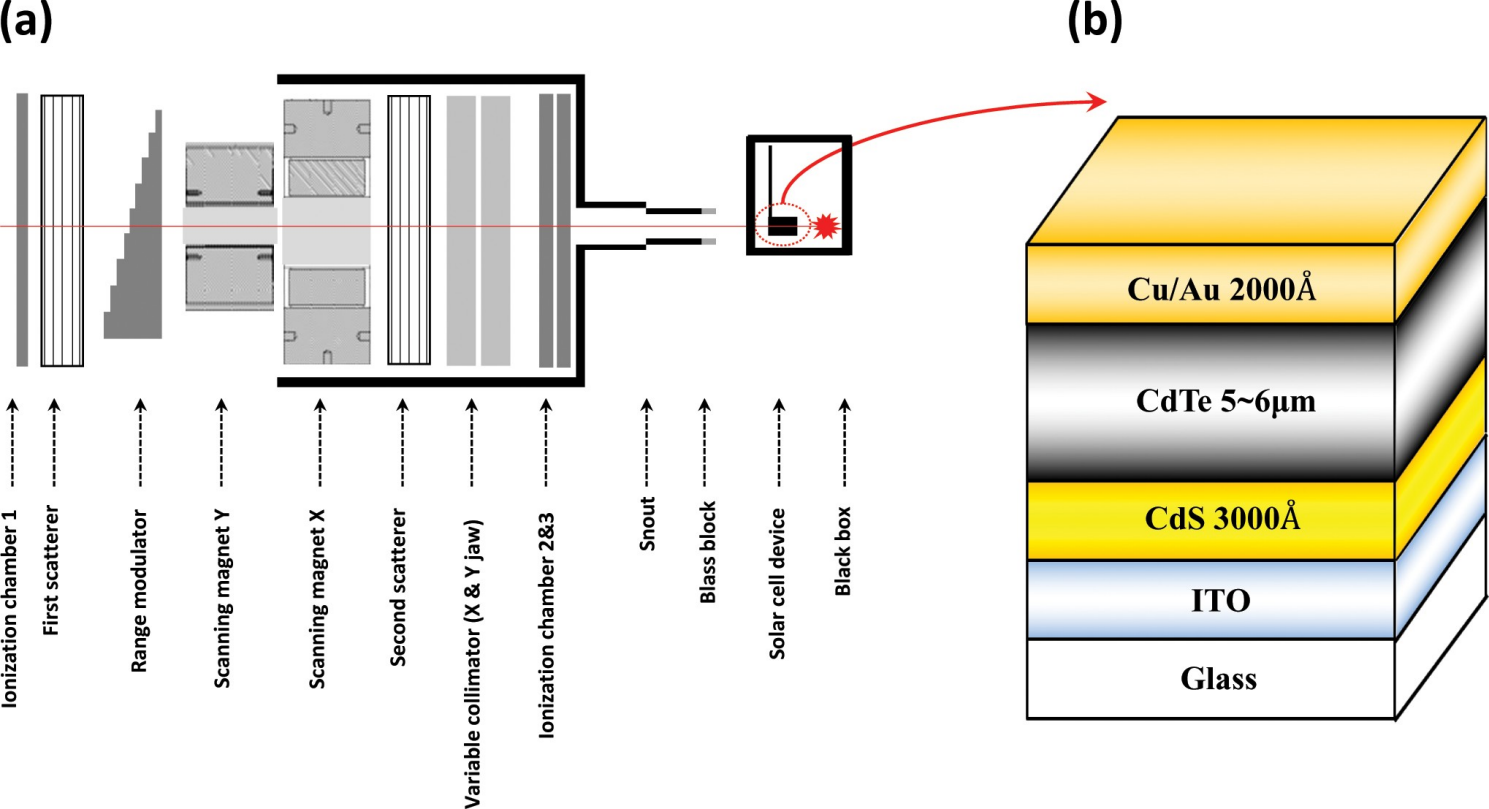
Schematics. (a) IBA UN at the NCC and (b) superstrate configuration of glass/ITO/CdS/CdTe/Cu/Au solar cell.

Fig 1A shows the universal nozzle (UN) of a proton therapy system (230 MeV, IBA Proton Therapy System-Proteus 235, Belgium) at the National Cancer Center (NCC) of Korea. The UN can achieve an appropriate trade-off between scattering mode, uniform scanning mode, and pencil beam scanning (PBS) mode and can use all three modes selectively. For this study, a 100 MeV proton PBS beam was irradiated at doses of 0, 10^12^, 10^13^, 10^14^, and 10^15^ cm^-2^. The CdTe devices, which were mounted on a homemade phantom composed of polymethyl methacrylate, were placed on PPS of a proton system in the direction of the glass substrate, which was also the direction of the PBS beam. Fifteen sister samples were prepared, and each PBS irradiation dose was applied to three samples and averaged. The equipment was calibrated at 1.2 MU‧cGy^-1^. To detect the signals during dosimetry sensing in a future study, it will be necessary to irradiate in the front direction owing to the characteristics of the solar cell.

### Fabrication of CdTe devices

The CdS/CdTe films were fabricated by applying a superstrate method to Corning 7059 glass coated with a 150-nm-thick indium tin oxide layer. The CdS films were deposited through thermal evaporation in a vacuum at a pressure of 10^−6^ torr using a direct current heating method and at a substrate temperature of 200°C and a deposition rate of 3 Å‧s^-1^. The high-purity CdS powder (99.995%, Sigma Aldrich) was used as the source material. The thicknesses of the CdS films were approximately 200–250 nm. The CdS samples were dipped into a saturated solution of CdCl_2_ in methanol and then annealed in air at 400°C for 30 min. The CdS interface is treated with CdCl_2_ to protect the CdS layer from the oxidation and to provide a suitable environment for the recrystallization of CdS particles. The CdTe films were also deposited onto the CdS samples using a high vacuum evaporation method at a substrate temperature of 350°C for 10 min. The deposition rate of the CdTe films was approximately 0.4–0.5 μm‧min^-1^. The resulting CdTe layers were 4.5–5.0 μm thick, with a compact morphology and a grain size of approximately 1 μm. The CdS/CdTe samples were dipped into a saturated solution of CdCl_2_ in methanol, then annealed in air at 400°C for 30 min. A 60-nm-thick Cu_2_Te layer was deposited onto the CdTe film at room temperature by evaporating a Cu_2_Te chunk source. The samples were subsequently annealed at 200°C for 10 min. To measure the performances of the fabricated samples mentioned above, Au films were deposited to a thickness of approximately 2,000 Å using DC sputtering at a working pressure of 10^−3^ torr as the top contact of the device structure. Fig 1B shows a schematic of the CdTe device.

### Characterization of device performance

After irradiation using PBS beams with various fluences, the performance of the CdTe cells was measured using current–voltage (J–V) characteristic analysis under AM 1.5 at 100 mW‧cm^-2^ illumination. External quantum efficiency (EQE) spectrum measurements were conducted using a measurement system including a monochromator and a xenon lamp. The spectral response of the solar cells was determined by measuring the photocurrent at each wavelength. The EQE was then calculated from the illumination intensity used as a fraction of the incident photons converted into an electrical current. Three samples, irradiated at doses of 10^12^–10^15^ cm^-2^, were labeled with their mean values and compared with the non-irradiated samples.

### Tool for particle simulation (TOPAS) test

**Fig 2:**
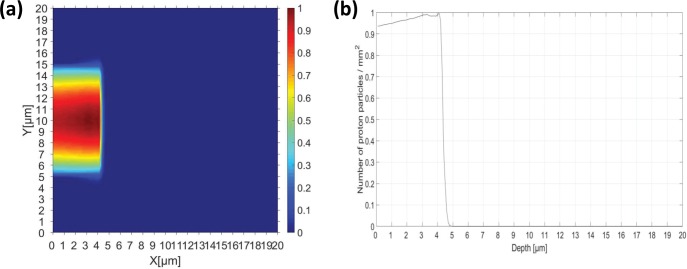
TOPAS simulation results. (a) Dose distribution and (b) fluence curves of hydrogen atoms in CdTe cells exposed to a 0.5 MeV PBS with a 1.5 cm diameter.

We conducted a Monte Carlo simulation to investigate how the hydrogen atoms generated by a proton beam interacted with the CdTe device according to the proton beam energy. A Monte Carlo simulation was performed using TOPAS 3.1. patch03 [20], which was used with circular mono-energetic beams with energies of 100, 120, 140, 160, and 200 MeV and diameters of 1.5 cm, during PBS [21]. We used physics data for proton beam therapy, as proposed by Zacharatou et al. [22], and information from the Geant4 material table to construct the material used in the CdTe detector. During the simulation, 1 × 10^7^ proton histories were used to calculate the dose and fluence deposited on the CdTe detector. Each simulation was conducted in parallel on two nodes with 56 core clusters using a computing cluster system (Intel Server Systems, Intel Corp, Santa Clara, CA, USA). As shown in Fig 2, to verify the simulation accuracy, we irradiated a 0.5 MeV PBS beam onto a CdTe solar cell to derive the (a) dose distribution and (b) fluence results for comparison with the results of Lamb et al. [12]. Fig 2 shows that the proton beam fluence completely penetrates the active layer and stops within the 5 μm region. We confirmed that the PBS beam system modeled using TOPAS was compatible with the Stopping and Range of Ions in Matter software used in previous studies.

## Results

Table 1 shows the numerical values of *V*_*oc*_, *J*_*sc*_, *FF*, and *ƞ*, which are the performance parameters of a photovoltaic cell assessed by measuring the *J–V* curve after irradiation by a PBS proton beam with an energy of 100 MeV and various dose fluences ranging from 1 × 10^12^ to 1 × 10^15^ cm^-2^. As shown in Table 1, the values of all of the parameters except for *V*_*oc*_ decrease as the fluence increases. The conversion efficiency, which is the main factor, also decreases from approximately 12% to 3% depending on the change in fluence. The measured parameter values were averaged by measuring three samples for each other proton fluence.

**Table 1:** Main parameters of photovoltaic cells at various fluences (including no irradiation) from 1×10^12^ to 1×10^15^ cm^-2^ averaged over three samples for each fluence. [a] indicates the difference in conversion efficiency before and after irradiation. [b] indicates the standard deviation for the performance parameters for all 15 samples before proton irradiation (3ea for each dose).

Dose	*V*_*oc*_		*J*_*sc*_		*FF*		*Ƞ*		*Ƞ*_dif. [a]_
(cm^-2^)	(mV)		(mA‧cm^-2^)		(%)		(%)		(%)
	Pre	Post	Pre	Post	Pre	Post	Pre	Post	
Ref.	760	759	22.2	22.1	64.3	64.4	12.0	12.0	0
1×10^12^	760	772	22.6	21.9	64.7	60.8	12.3	11.5	-0.8
1×10^13^	755	761	21.2	20.9	64.4	57.5	11.9	10.1	-1.8
1×10^14^	780	738	22.4	20.8	65.4	53.4	12.5	8.4	-4.1
1×10^15^	755	673	21.4	9.4	61.4	42.4	11.6	3.4	-8.2
σ^[b]^	9.00	-	0.56	-	1.37	-	0.31	-	-

**Fig 3:**
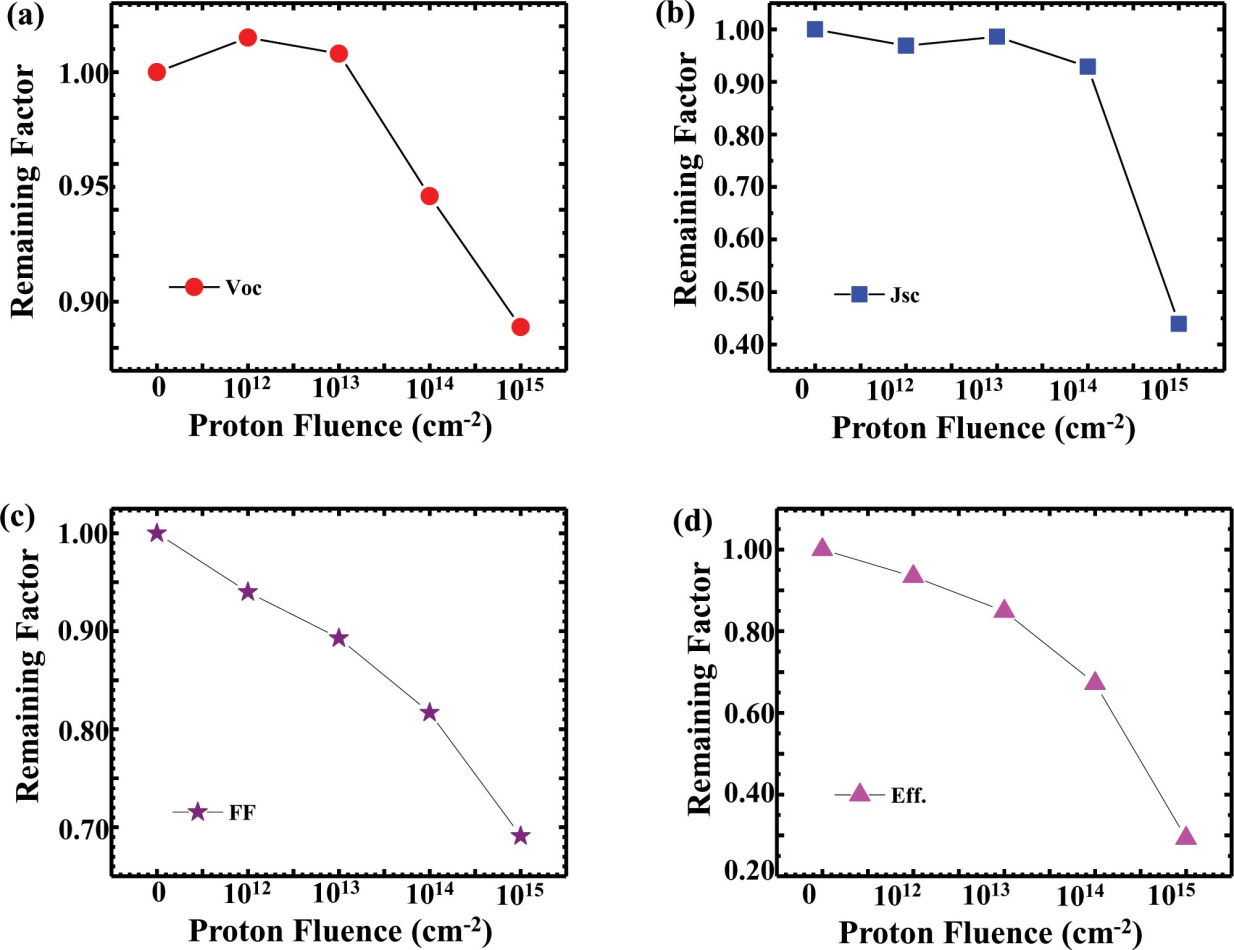
Remaining factors, showing the degradation according to fluence. (a) *V*_*oc*_, (b) *J*_*sc*_, (c) *FF*, and (d) *ƞ* in CdTe solar cells irradiated with a 100 MeV PBS beam.

Fig 3 shows the degradation of *V*_*oc*_, *J*_*sc*_, *FF*, and *ƞ* in CdTe solar cells irradiated with a 100 MeV PBS beam according to fluence. To assess the degradation mechanism of each parameter, the remaining factor—defined as the ratio of the degraded value to the initial value—is shown as a function of the proton fluence in Fig 3. In Fig 3A, *V*_*oc*_ exhibits gradual degradation with changing fluence, increasing slightly when the fluence is 1×10^12^ cm^-2^ and decreasing to 89% at a fluence of 1×10^15^ cm^-2^.

There are several reasons for changes in *V*_*oc*_:

1. *V*_*oc*_ is affected by the formation of ohmic contacts depending on the doping concentration of the back-contact material at the CdTe/metal interface. In this study, the hydrogen atoms implanted by the proton dose passivated the copper, resulting in a lower doping concentration.
2. Owing to the heat generation induced through proton irradiation, copper is excessively diffused, damaging the junction, or sulfur and telluride are overly intermixed, resulting in a minority carrier lifetime [23].

**Fig 4:**
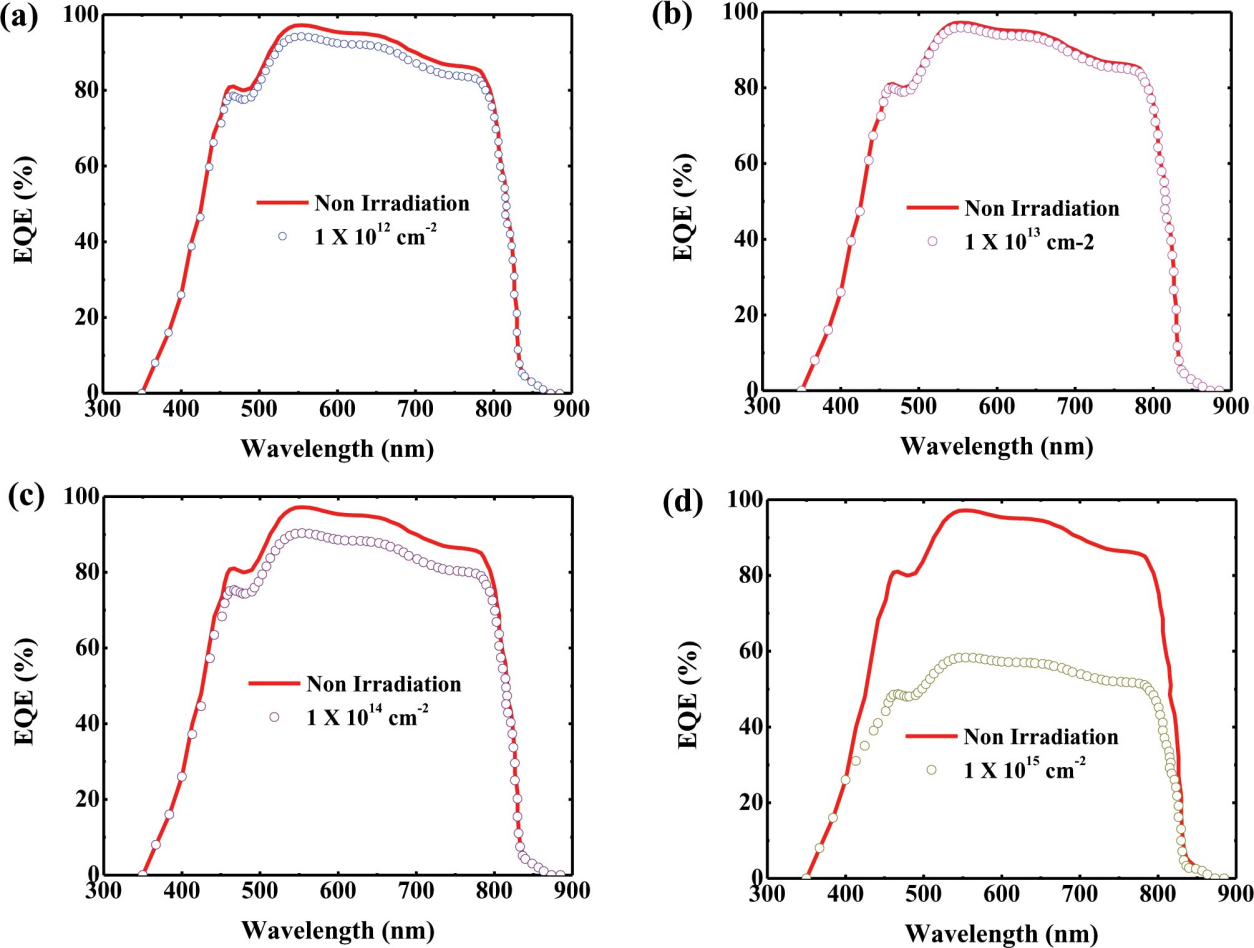
EQE spectra of CdTe cells with various proton doses. The EQE was measured after irradiation with a PBS beam at fluences of (a) 1×10^12^ cm^-2^, (b) 1×10^13^ cm^-2^, (c) 1×10^14^ cm^-2^, and (d) 1×10^15^ cm^-2^ and compared with that of a non-irradiated CdTe cell.

Fig 3B shows the degradation of *J*_*sc*_ as the proton dose changes. *J*_*sc*_ changes slightly when the fluence increases from 1×10^12^ to 1×10^14^ cm^-2^ but decreases to 44% of that of the reference cell at the highest proton dose of 1×10^15^ cm^-2^. The change in *J*_*sc*_ as a function of the proton dose is thought to be due to a decrease in the minority carrier lifetime, as mentioned in regard to *V*_*oc*_ section. In the case of *V*_*oc*_, the effect was almost at the junction, whereas the drop in *J*_*sc*_ is attributable to trapping of the carriers generated at the junction in the recombination center formed by the hydrogen atoms in the entire CdTe layer. In addition, *J*_*sc*_ decreased because the incident light could not penetrate more than a certain amount owing to the darkening of the borosilicate glass caused by proton irradiation [11]. The degradation mechanism of *J*_*sc*_ is further illustrated by the EQE data shown in Fig 4.

Fig 3C and 3D show the degradation of *FF* and *ƞ*, respectively, according to the proton fluence, demonstrating that the remaining factors decrease linearly with increasing fluence. For the highest proton dose, *FF* and *ƞ* decrease by 69% and 29%, respectively, compared to those of the reference cell. The conversion efficiency, in which *V*_*oc*_, *J*_*sc*_, and *FF* are combined, dramatically decreases with respect to the maximum dose, whereas at a proton dose of 1×10^14^ cm^-2^, the conversion efficiency remains at approximately 70% of that of the reference cell.

In this study, the conversion efficiency of the CdTe solar cell at the highest proton dose was degraded to approximately 30% of that of the reference cell, and the most influential parameter was *J*_*sc*_, as can be seen in Fig 3. For better understanding of the degradation process of *J*_*sc*_, the EQE spectra of CdTe cells at various proton doses are shown in Fig 4. The spectral response of the photovoltaic cells was obtained at each wavelength, and the EQE was calculated from the illumination intensity used as the fraction of incident photons converted into an electrical current [24]. As shown in Fig 4, as the fluence of the proton beam increases, the EQE tends to decrease gradually. The EQE is slightly increased at a proton fluence of 1×10^13^ cm^-2^, which is in good agreement with the results shown in Fig 3B. As shown in Fig 4C, the EQE is approximately 90% of that of the reference sample at a fluence of 1×10^14^ cm^-2^, and the corresponding *J*_*sc*_ is approximately 93% of that of the reference cell, as shown in Fig 3C. Figs 3D and 4D demonstrate that the *J*_*sc*_ and EQE are 44% and 60%, respectively, of the corresponding values for the reference sample at a maximum dose of 1×10^15^ cm^-2^. As shown in Fig 4D, the EQE is degraded by more than 40% within the wavelength region of approximately 400–800 nm. The degradation at 500–800 nm, which is the absorption range of the solar cell according to the band gap of CdTe, is due to a decrease in the minority carrier lifetime owing to the hole trapping occurring at the recombination center, as depicted in Fig 3. However, the decrease in the EQE at 400–500 nm is thought to be due to the darkening phenomenon of glass during proton irradiation. This darkening phenomenon occurs when a proton beam is irradiated over a certain amount and is barely observable at fluences other than the highest one. We also found that the EQE is good for devices affected by proton irradiation at all wavelengths, and the EQE peaks begin to decrease dramatically at a wavelength of 800 nm near the band edge of the CdTe.

### TOPAS simulation and discussion

**Fig 5:**
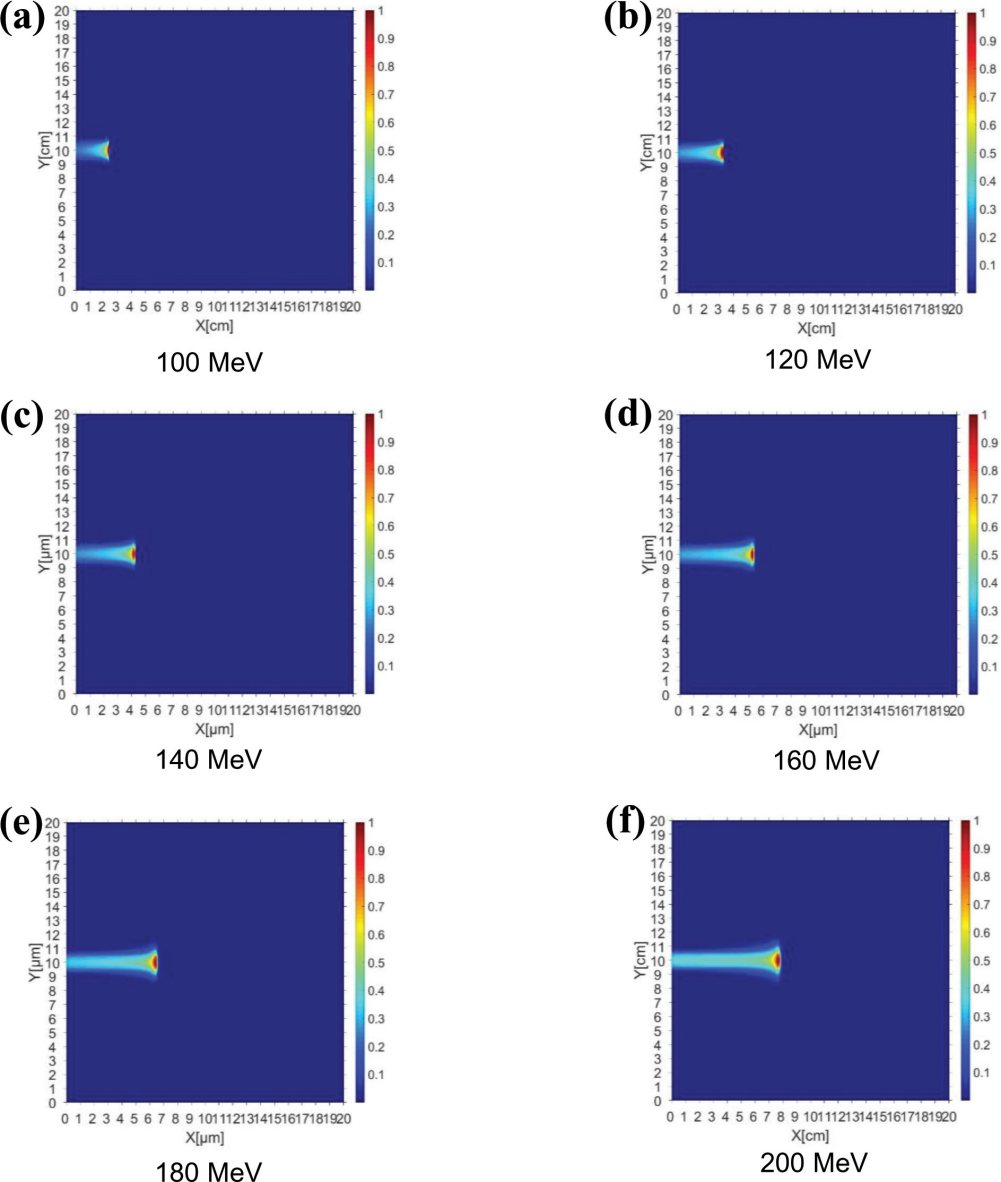
TOPAS-based simulation results. Proton dose distributions according to layer depth obtained by the trajectories of PBS proton beams with energies of (a) 100, (b) 120, (c) 140, (d) 160, (e) 180, and (f) 200 MeV.

Fig 5 presents the results of a simulation of the proton dose distribution according to the layer thickness obtained by the trajectory of the proton beam when a 100–200 MeV PBS proton beam was irradiated onto a CdTe solar cell using the TOPAS program. It was assumed that there was a cover glass of sufficient thickness to allow the incident protons to be blocked. As shown in Fig 5, the penetration depth of the protons varied from 2.3 to 7.6 cm when irradiation was performed using a proton beam energy of 100–200 MeV. This finding implies that the irradiated proton beam passed through the entire CdTe cell layer and that the ionized energy was significantly less because it interacted little with the other materials. Therefore, damage due to proton irradiation will be distributed throughout the CdTe device. Because most of the damage was concentrated near the penetration depth owing to the characteristics of the Bragg peak of the proton beam, as shown in Fig 5A–5F, the dose distribution for all energies is concentrated at the end of the projected range. Therefore, the damage to the photovoltaic cell by proton irradiation is determined by the amount of proton fluence accumulated in the active layer depending on the energy level because the proton energy passes through the total layer when the proton energy is more than 0.5 MeV.

**Fig 6:**
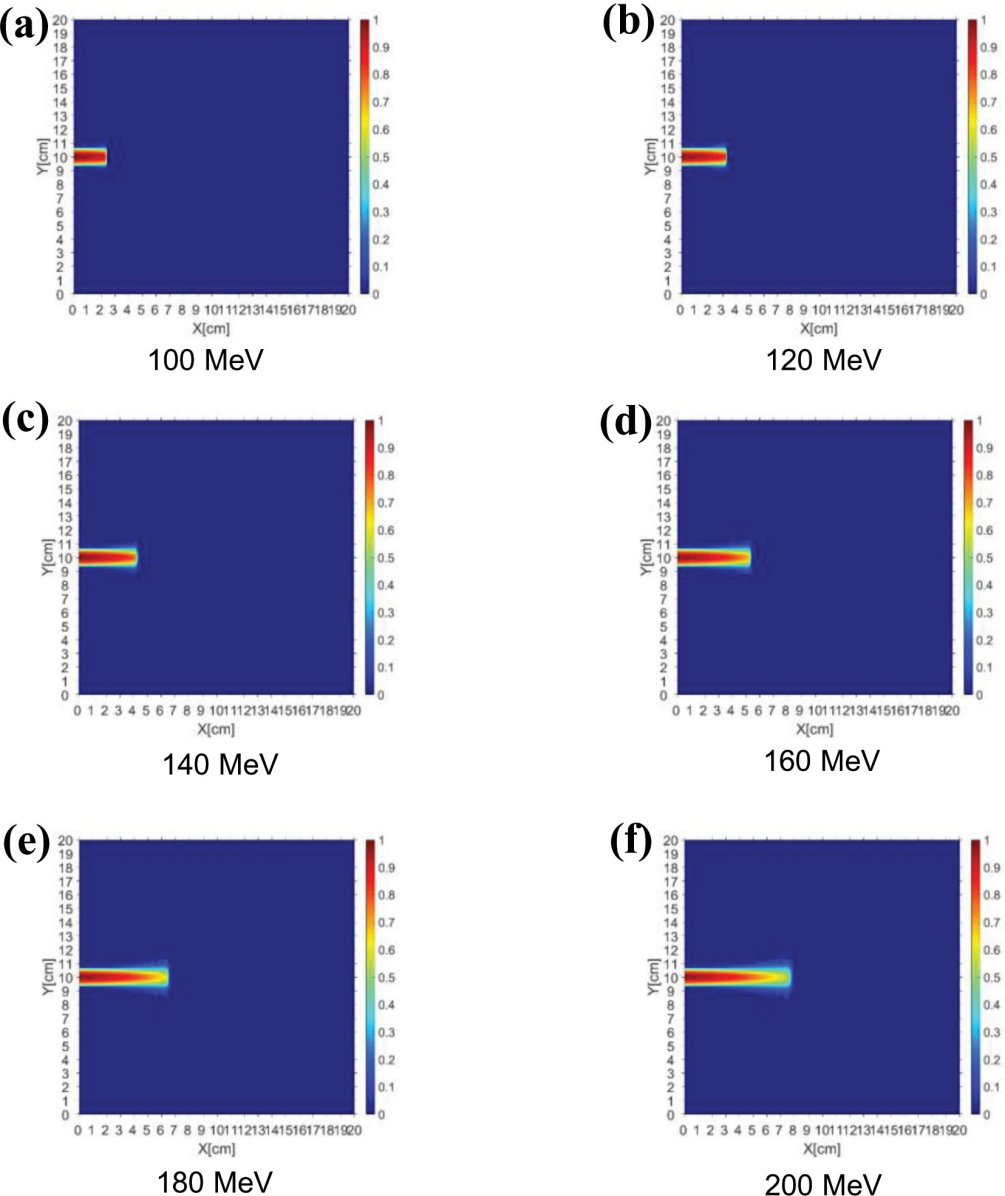
Dose distributions of accumulated protons. Dose distributions obtained using PBS proton beams of the same fluence and energies of (a) 100, (b) 120, (c) 140, (d) 160, (e) 180, and (f) 200 MeV.

Fig 6 shows the quantity of protons accumulated in the cell depending on the energy level when PBS proton beams of the same fluence and energies of up to 100–200 MeV were irradiated onto the CdTe cell. As shown in Fig 6, as the proton energy increases, the drop rate of the hydrogen fluence decreases. Owing to the characteristics of the Bragg peak of a proton, most of the fluence is consumed near the beam range; thus, a higher fluence will accumulate at a smaller thickness with a lower energy beam than with a higher energy beam [4]. In other words, it is likely that the number of hydrogen atoms that accumulate in the active layer when the same fluence is applied to the cell will be high. In the case of a CdTe cell, because the thickness of the entire layer is less than 10 μm, the amount of fluence accumulated at each energy level is almost the same. However, when the cover glass is fabricated for the space mission of a solar cell, the fluence distribution at each proton beam energy should be analyzed as a function of the thickness, and this study has an important meaning.

In this study, the effects of proton radiation on solar cells were investigated by irradiating 100 MeV PBS protons onto a CdTe cell. A conversion efficiency of approximately 70% of that of the reference cell (without irradiation) was maintained even when a fluence of 1×10^14^ cm^-2^ was applied. In addition, owing to the Bragg peak characteristics of the protons, the higher the energy, the less fluence accumulates in the photovoltaic cell and the less damage the cell receives. Of course, because the total layer thickness of the solar cell is extremely small and the amount of accumulated fluence is stochastic, the amount of damage will not change much as the applied energy is varied; thus, further study is necessary.

This work also serves as a preliminary study for the application of solar cells as dosimetry sensors. As mentioned in the introduction, there has been a continual effort to replace existing dosimetry tools. However, several problems are expected in signal detection when actual commercialization is achieved. Therefore, to enable the use of solar cells as dosimetry tools in proton therapy mode, we investigated the effects of radiation on CdTe solar cells at energies corresponding to proton treatment beams, i.e., 100 MeV and higher. As a result, we confirmed the feasibility of developing a dosimetry tool consisting of CdTe devices by analyzing the degradation of CdTe cells caused by proton irradiation. As the TOPAS simulation demonstrated, the energy of a proton beam is so large that it completely penetrates the CdTe cell, making it is difficult to form an electric signal directly through the absorption of generated carriers during irradiation.

In a previous study, we developed an acrylic disk radiation sensor system that could calculate the range by measuring the Bragg peak according to the energy of the proton PBS beam using an acrylic disk [25]. Detecting the photoluminescence signal generated by a disk with an optical fiber results in considerable signal loss and complex detection process. Thus, it is necessary to detect the generated signal directly by applying a solar cell to a disk. Therefore, this study is essential to confirming the damage of solar cells in 100 MeV proton therapy beams. In addition, in a previous study, Kang et al. used a metal plate on a CdTe film to detect a 6 MV photon beam and converted the photon beam into a secondary electron beam to observe an electrical signal [14]. We are working on an experiment based on a similar concept by applying a scintillation film that can transfer the proton energy to the solar cell and convert the proton energy into secondary electron energy or an electrical signal.

## Conclusion

We investigated the effects of radiation by a proton therapy beam on CdTe-based solar cells by irradiating a proton PBS beam with 100 MeV of energy. As a result, *V*_*oc*_, *J*_*sc*_, and *FF*, which are the main parameters of the solar cell, were approximately 89%, 44%, and 69%, respectively, of the corresponding value for the reference cell (without irradiation) at the highest dose of 1×10^15^ cm^-2^. In particular, *ƞ* was reduced to approximately 30% of its reference value owing to the combined effects of the parameters. The CdTe cell recorded an efficiency rate of approximately 70% even at a high fluence of 1×10^14^ cm^-2^, confirming the durability of CdTe solar cells for space missions and their future application as dosimetry sensors. Likewise, a TOPAS-based simulation showed that the proton fluence curves accumulated in a CdTe cell when irradiated with a 100–200 MeV proton beam. Based on the Bragg peak characteristics of protons, we assessed the relatively high proton fluence at low energies compared to that at high energies. The high probability of incurring more radiation damage after low-energy irradiation was also addressed. This work serves as a preliminary study for the application of thin-film CdTe-based solar cells in proton therapy beam mode and is expected to provide valuable reference material for studies on thin-film solar cell proton dosimetry sensors. To improve the performance of the CdTe cell, we have studied a CdCl_2_ treatment method using Freon gas, and aim to apply it to CdTe cell fabrication. Studies are being conducted to achieve reproducible performance, while reducing the thickness of CdS and CdTe. To increase the durability, a new surface treatment method in CdTe/metal interface is also being studied.
